# Optimizing skull base osteomyelitis treatment: effectiveness of antibiotic therapy within a multimodal approach

**DOI:** 10.1007/s00405-025-09915-7

**Published:** 2025-12-22

**Authors:** Miriam Simon, Mara Wichtrup, Laurenz Althaus, Insa Joost, Sven Dreyer, Christian Rubbert, Jörg Schipper, Julia Kristin

**Affiliations:** 1 Department of Otorhinolaryngology, Duesseldorf University Hospital, Moorenstrasse 5, 40225 Duesseldorf, Germany; 2 Department of Medical Microbiology and Hospital Hygiene, Duesseldorf University Hospital, Moorenstrasse 5, 40225 Duesseldorf, Germany; 3 Department of Orthopedics and Trauma Surgery, Duesseldorf University Hospital, Moorenstrasse 5, 40225 Duesseldorf, Germany; 4 Department of Diagnostic and Interventional Radiology (Neuroradiology), Duesseldorf University Hospital, Duesseldorf, Germany

**Keywords:** Skull base osteomyelitis, Skull base surgery, Hyperbaric oxygen therapy, Antibiosis, Multimodal approach

## Abstract

**Purpose:**

Skull base osteomyelitis (SBO) is a potentially life-threatening condition requiring multimodal treatment, including antibiotic therapy, surgery, and hyperbaric oxygen therapy. This study aimed to evaluate the role and the effectiveness of antibiotic therapy in the management of SBO.

**Methods:**

A retrospective analysis was conducted on 52 adult patients treated for SBO at the Department of Otolaryngology between 2006 to 2025. The study focused on the selection, duration, and mode of antibiotic administration, guided by microbiological findings. Antibiotic regimes were tailored based on pathogen sensitivity, surgical intervention and hyperbaric oxygen therapy provided as indicated.

**Results:**

Of the 52 patients, 75.0 % were male and 25.0 % female. The mean age was 70.6 years (SD=11.0). A total of 301 swabs and biopsy samples were collected; in 11.5 % of cases, pathogens were identified only via biopsy. Despite repeated sampling, no pathogen was found in 19.2 % of patients. One patient required 20 samples before *Mycoplasma* was identified. The average duration of intravenous antibiotics was 31.9 days. Surgery was performed in 71.2 % of patients, and 55.8 % received hyperbaric oxygen therapy.

**Conclusion:**

Pathogen-specific antibiotic therapy following tissue sampling is a cornerstone of SBO treatment. Accurate microbiological diagnosis and individualized antibiotic regimens are essential for effective multimodal management.

## Introduction

Skull base osteomyelitis (SBO) is a rare but serious infection of the temporal bone, associated with high morbidity and mortality. But what exactly is this disease? 

According to current literature, SBO is most commonly classified into two categories: typical and atypical skull base osteomyelitis [1–4]. Typical SBO (TSBO) is more prevalent and primarily affects elderly patients with diabetes mellitus. It commonly arises as a complication of malignant otitis externa, most often caused by *Pseudomonas aeruginosa*. In contrast, atypical SBO (ASBO)-also referred to as central SBO-primarily involves the sphenoid and occipital bones and generally occurs without preceding otitis [5].

Given the potentially life-threatening nature of this disease, timely and effective treatment is critical. Ideally, patients should be managed in specialized skull base centers where care is delivered through a multidisciplinary approach. Treatment strategies are complex and tailored to the individual patient, typically involving the three established pillars of therapy: surgical intervention, antibiotic therapy, and hyperbaric oxygen therapy.

Antibiotic treatment is a cornerstone of SBO management. To appreciate its impact, it is worth recalling the transformative role of antibiotics in modern medicine. The discovery of penicillin by Sir Alexander Fleming in 1928 revolutionized the treatment of bacterial infections. Since then, numerous other antibiotics have been developed, significantly improving outcomes in infectious diseases [6].

In the context of SBO, empiric antibiotic therapy is essential. Initial regimens often include broad-spectrum antibiotics, such as piperacillin-tazobactam, ceftriaxone, or cefotaxime, frequently combined with metronidazole to ensure anaerobic coverage [7]. Once culture and sensitivtiy results are available, antibiotic therapy should be adjusted accordingly. In cases involving *P. aeruginosa* infection, ciprofloxacin or ceftazidime may be appropiate, though emerging resistance patterns must be carefully considered [7].​ The duration of antibiotic treatment typically ranges from several weeks to months, depending on the severity of infection and clinical response. In some cases, surgical debridement may be necessary to remove necrotic bone tissue. Notably, hyperbaric oxygen therapy has been shown to exert effects comparable to intravenous antibiotics, by enhancing oxygen delivery and promoting wound healing.

Since 2018, our institution has implemented a Antibiotics Stewardship Program (ASP), which provides consultative support to optimize antimicrobial use. These programs aim to improve patient outcomes, minimize adverse effects, and combat the rise of antibiotic resistance. ASPs have become standard pratice globally, particularly in larger, academic and university hospitals. While implementation may vary depending on national resources and infrastructes, international awareness and adherence to stewardship guidelines are steadily increasing. Notable examples of successful ASP frameworks have been reported in Europe as well as in the United States, Australia, India, and several Latin American countries [8].

The aim of this study was to evaluate the type and role of antibiotic therapy in the treatment outcomes of patients with SBO. With a wide array of antibiotics available, selecting the appropriate agent, dosage, duration, and route of administration is essential and must always be pathogen specific. Treatment response is monitored through both clinical assessment and radiologic imaging.

## Materials and methods

A retrospective analysis was conducted on all patients treated for SBO (TSBO and ASBO) at the Department of Otolaryngology in our hospital from 2006 to 2025. The study included *n* = 52 patients aged 18 years or older, who were categorized into two groups: typical and atypical skull base osteomyelitis. A comprehensive dataset was compiled, detailing the number, type, duration, and any changes in the antibiotics administered. Additionally, patient outcomes were thoroughly evaluated based on clinical and radiological findings.

Approval was obtained from the local ethics committee (Approval No: 2024–2939).

### Antibiotic therapy

The type of antibiotic, dosage, duration, route of administration, and any potential adverse effects were evaluated.

### Clinical symptoms

Descriptive data and clinical symptoms of the 52 patients, including cranial nerve deficits, were recorded. Additionally, the results of microbiological analyses were collected and analyzed.

### MR and CT imaging

MRI was included in the analysis as both baseline and post-treatment scans were available. All images were assessed by the local neuroradiology department, focusing on the extent of the skull base osteomyelitis. MRI was performed including contrast enhanced using a 3D T1-weighted MPRAGE sequence (TR = 2.0s, TE = 2.45ms, TI = 900ms, flip angle = 8°, field of view = 250 × 250 mm, slices = 160, voxel size = 1 mm³). The imaging evaluation focused on the extent of inflammation and degree of contrast enhancement. Computed tomography (CT) was performed as needed during treatment to further assess bony involvement, and particularly to assess re-sclerosis on follow-up imaging.

### Statistics

Microsoft Excel (Version 16.81) and IBM SPSS Statistics (Version 29) were used for the statistical analysis of the dataset.

## Results

### Patient characteristics

A total of *n* = 52 patients treated with skull base osteomyelitis at our clinic were included. Of these, 75.0% were male and 25.0% were female. The mean age at the time of initial diagnosis was 70.6 years (SD = 11.0). As a certified skull base center equipped with a hyperbaric oxygen therapy chamber, our university hospital serves as a referral center for complex cases. More than half of the patients (55.8%) were referred from other hospitals, while 44.2% presented from home, either through our outpatient clinic or via the central emergency department. No patients were admitted from nursing homes.

In the present cohort, 88.5% of patients were diagnosed with typical skull base osteomyelitis. Consequently, most patients presented with symptoms such as otalgia and otorrhea; in some cases, particularly those with advanced disease, cranial nerve deficits involving the facial nerve or the caudal group were already evident (see below).

A positive smoking history was documented in 20.5% (*n* = 8/39) of patients. Notably, the prevalence of diabetes mellitus was high, affecting 61.5% of the cohort. This form of diabetes was characterized by atypical HbA1c values, necessitating co-management by endocrinology specialists. Additionally, 57.7% of patients had cardiovascular disease requiring medical treatment. A history of ear, nose, and throat tumor was identified in 2 of the 52 patients. Three of the patients were immunosuppressed due to an underlying haematological malignancy. One patient has undergone radiation therapy for squamous cell carcinoma of the ear canal, which has also spread to the lateral skull base.

### Clinical findings

Cranial nerve palsies were observed in 61.5% of patients, with facial nerve palsy being the most common, present in 40.4% at baseline. During treatment, partial to complete regression of facial nerve palsy was documented in 55.5% of cases (*n* = 10/18, with missing documentation). Due to anatomical proximity, caudal cranial nerves were also affected in a subset of patients. In this cohort, 30.8% presented with paresis of at least one caudal cranial nerve at baseline. During treatment, 46.2% (*n* = 6/13, with missing documentation) showed partial to complete improvement. In three patients, the clinical course of the paresis could not be evaluated due to death (*n* = 6 total deaths).

Additionally, 11.5% of patients presented with paresis of at least one other cranial nerve at baseline. Among these, 5 out of 6 cases involved abducens nerve (cranial nerve VI) palsy, with 3 out of 5 showing partial to complete recovery during treatment. One case involved oculomotor nerve (cranial nerve III) palsy, although no documentation was available regarding its progression or resolution. In two patients (*n* = 2/6), the affected cranial nerve manifested as neuropathy or ophthalmoplegia.

Elevated C-reactive protein (CRP) levels were observed in 81.3% (*n* = 39/48) of patients at the start of treatment or upon hospital admission (norm: CRP < 0.5 mg/dl). In contrast, leukocytosis was present in only 14.3% (*n* = 7/49) of patients (norm: leukocytes x1000/microliter 3.9–10.9).

### Biopsy and Swab-based diagnostic

Across the entire cohort, a total of *n* = 301 swabs and biopsy samples were collected, with multiple specimens often obtained per patient. Of these, 155 were swabs and 146 were tissue or biopsy samples. In 11.5% of cases (*n* = 6), bacterial pathogens were identified only after obtaining a biopsy sample rather than a swab. In one patient, up to *n* = 20 samples were collected across multiple hospital admissions before the correct pathogen was ultimately identified, where mycoplasmas were finally detected. Despite repeated sampling and biopsy, 19.2% of patients showed no microbiological evidence of a pathogen. A biopsy was also performed to exclude the possibility of a malignant tumor, which can sometimes present with similar symptoms. None of the biopsies in this cohort demonstrated the presence of a malignancy.

**Fig. 1:**
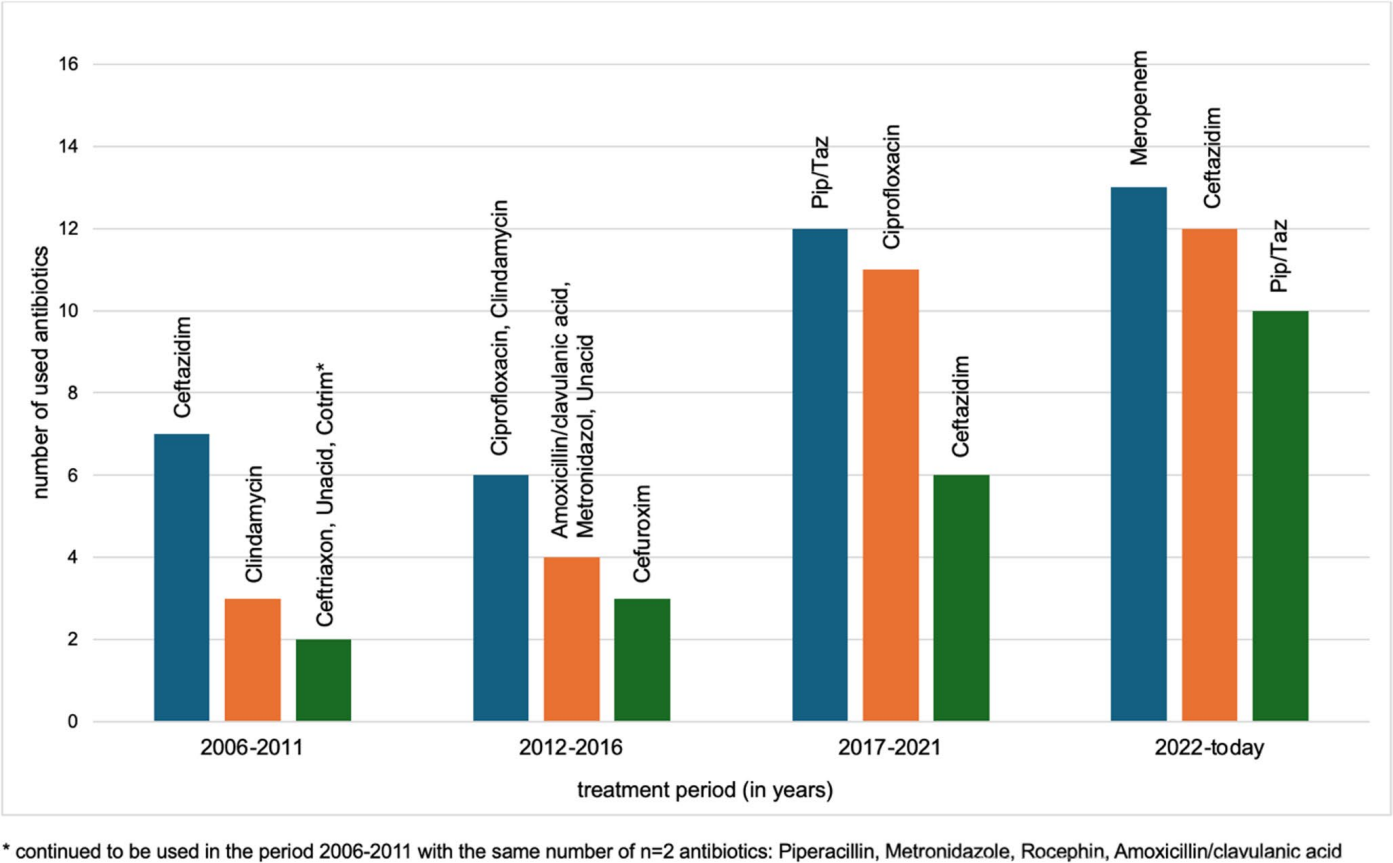
An analysis of the evolution in the administration of antibiotics over time in our university clinic

**Fig. 2:**
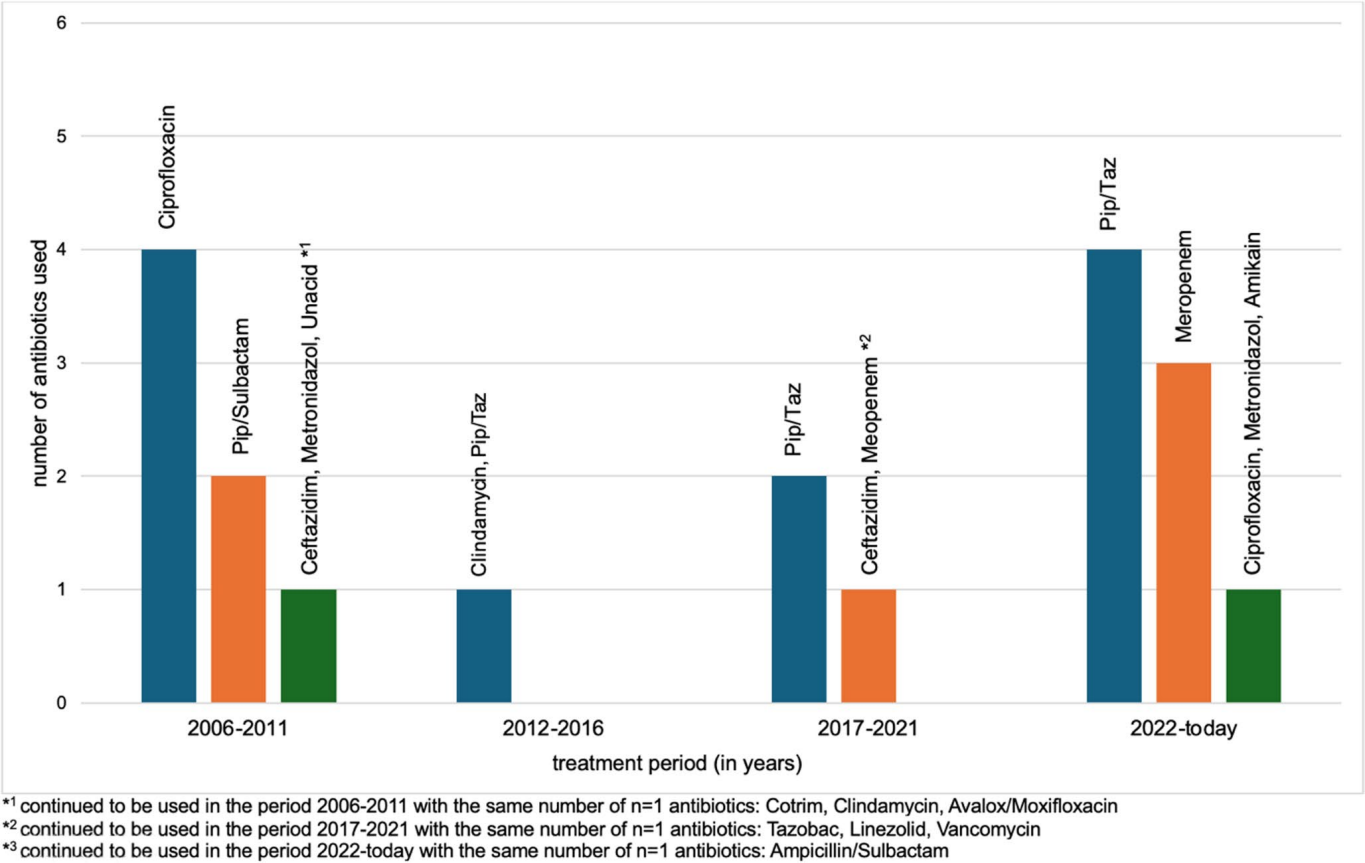
An analysis of the evolution in the administration of antibiotics over time in external hospitals

Additionally, trends in antibiotic prescribing practices since 2006 were analyzed, both within our institution and in referring hospitals. Over time, there has been a noticeable increase in the consistent use of piperacillin/tazobactam in more recent cases, particularly those involving *Pseudomonas aeruginosa*. Use of meropenem and ceftazidime has also increased, reflecting their effectiveness against gram-positive cocci (e.g., *Staphylococcus aureus*) and *Pseudomonas aeruginosa* (see Figs. 1 and 2).

### Antibiotic therapy

The average duration of intravenous antibiotic treatment was 31.9 days (SD = 22.6, range: 0 to 84 days). Regarding the minimum duration, one patient was referred solely for HBO therapy from an external hospital, but the specific antibiotics administered prior to referral are unknow. Two patients received antifungal therapy. Additionally, 78.9% of patients underwent multiple courses of antibiotics, and 44.2% continued oral antibiotic therapy after hospitalization, in some cases extending beyond 25 weeks.

As part of the multimodal treatment approach, 55.8% of patients received hyperbaric oxygen therapy, and 71.2% underwent surgery. All patients were offered hyperbaric oxygen therapy. However, some refused, while others were in too poor a state of health. Surgical interventions ranged from intraoperative sampling alone to extensive surgical debridement of the mastoid and/or paranasal sinuses.

### Imaging

In addition to clinical symptoms and laboratory parameters, CT and contrast-enhanced MRI imaging were used to assess the exent of inflammation. Initial imaging was performed to confirm the diagnosis, either at our institution or elsewhere. Follow-up imaging was conducted after completion of treatment, with additional scans typically performed at approximately 6 months. A final imaging assessment was performed at 12 months, depending on earlier findings.

At around 3 months, 30.0% of patients (*n* = 40, with missing data) demonstrated regression of inflammation, 12.5% showed no change, and 57.5% showed progression. Notably, no patient exhibited complete resolution at this time point. At 6 months, among 27 patients evaluated, 37.0% showed regression, 11.2% showed no change, and 48.1% showed progression. A cure was documented in 3.7% of patients at 6 months. At 12 months, 80.8% of the patients (*n* = 26) showed regression, 7.7% no change and 7.7% progression, with a documented cure rate of 3.8%.

36.5% (*n* = 19/52) were readmitted up to two years following their initial diagnosis due to the worsening of clinical symptoms and progression on MRI.

**Fig. 3:**
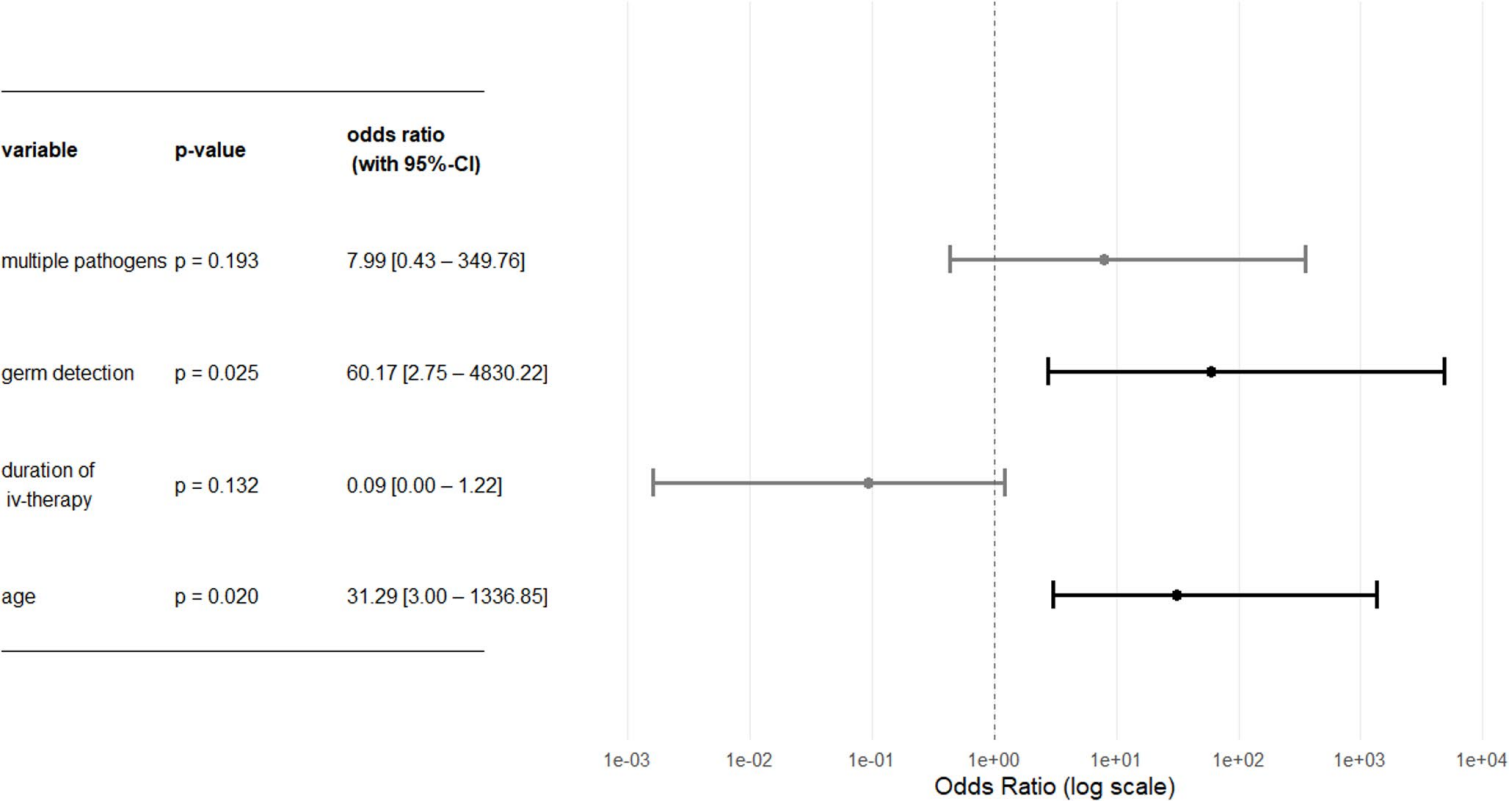
Significance between mortality risk and age/germ detection

Statistical analyses revealed a statistically significant correlation between patient age and mortality risk in skull base osteomyelitis (*p* = .02), as well as a correlation between mortality risk and pathogen detection (*p* = .03) (see Fig. 3).

## Discussion

Skull base osteomyelitis is a rare but potentially life-threatening condition, with reported mortality rates reaching up to 50.0% [9]. Data regarding the optimal management are scarce and national or international guidelinies advising the optimal antibiotic treatment and duration of therapy are lacking. Management is particularly challenging in elderly patients, those with multiple comorbidities, and in cases where microbiological identification of the pathogen is not possible. It highlights the necessity of a multifaceted treatment approach to SBO, underscoring the critical role of timely and appropriate antibiotic therapy-even in the absence of a confirmed pathogen [10, 11].

In the setting of a certified skull base center such as ours, regular interdisciplinary discussions and ongoing clinical evaluation are essential. The clinical presentation of skull base osteomyelitis is complex and often multimodal, necessitating an equally multimodal therapeutic strategy. As surgeons, we greatly benefit from the close collaboration with the Antibiotic Stewardship team, whose guidance and expertise are invaluable in optimizing treatment regimes. Effective therapy requires a collaborative partnership between clinicans and patients.

Given the predominance of patients over the age of 70 in our cohort, it is crucial to consider comorbidities alongside the antibiogram when selecting appropriate antimicrobial therapy. For example, in patients with impaired renal function, close clinical moniotoring and laboratory assessment are essential when administering potentially nephrotoxic agents such as gentamicin or vancomycin [12].

However, implementing strictly pathogen-directed antibiotic therapy has proven to be more challenging than initially anticipated. In our cohort, 55.8% of patients were transferred from other institutions-often due to inadequate clinical response to prior treatmet or lack of access to advanced therapies such as hyperbaric oxygen therapy. In many of these cases, empirical antibiotic therapy had already been initiated prior to transfer, complicating subsequent pathogen detection. As a result, no pathogen could be identified in 19.2% of patients despite the collection of multiple specimens and the presence of clear clinical, laboratory, and radiologic indicators of SBO. While this rate is lower than figures reported in the literature-where up to 30.0% of SBO cases show no microbiological evidence of infection it remains clinically significant [13].

This phenomenon, though relatively uncommon, affects roughly one in five patients in real-world settings and underscores the need for careful interdisciplinary review and consultation with the antibiotic stewardship team.

Clear recommendation for the optimal empiric antibiotic treatment is not available, however, in cases where no pathogen is identified despite extensive culturing and repeated sampling, the initial treatment strategy should include *Pseudomonas aeruginosa* as well as *Staphylococcus aureus*, both frequently associated pathogens in skull base osteomyelitis. Empiric therapy might be initiated with high-dose piperacillin/tazobactam at a dose of 4.5 gram intravenously every 6 h, which also provides coverage against other commonly implicated organism. If methicillin-resistant *Staphylococcus aureus* (MRSA) is suspected, additional vancomycin or, if a blood stream infection is excluded, linezolid (600 mg orally or IV every 12 h) may be used as a treatment option. Regular clinical reassessment is imperative for all patients with confirmed or suspected infection bearing in mind that systemic inflammatory markers like leucocytes or C-reactive peptide usually are not helpful. For those without definitive evidence of infection, reevaluation remains a priority to ensure appropriate antimicrobial stewardship and guide ongoing management.

In analogous clinical situations, other medical specialties employ similarly structed therapeutic strategies. A comprehensive review of the current literature reveals clear parallels, supporting the effectiveness of the multimodal treatment approach implemented in skull base osteomyelitis [14]. Examples of compared therapeutic interventions include: (1) Post-traumatic or post-surgical Osteomyelitis [15]: Treatment often involves a combination of a second- or third-generation cephalosporin with clindamycin, or an aminopenicillin combined with a beta-lactamase inhibitor. In cases of staphylococcal infection, monotherapy with flucloxacillin or a first-generation cephalosporin is preferred. (2) Diabetic Foot Osteomyelitis:[14] Empirical therapy typically includes vancomycin (0.5–2.0 g/day IV) in combination with piperacillin-tazobactam (4.5 g IV every 6 h). These parallels underscore the relevance of broad-spectrum initial therapy in osteomyelitis conditions where pathogen identification is delayed or unsuccessful, followed by targeted de-escalation based on clinical and microbiological response.

It is evident that a common principle underlines all three therapeutic approaches: The optimal duration of antibiotic therapy generally ranges from 4 to 6 weeks, depending on clinical improvement, radiologic resolution, and normalization of inflammatory markers. ​Transition to oral antibiotics may be appropriate if the patient is clinically stable, adequate source control has been achieved, and gastrointestinal absorption is not impaired [14].

Other specialties also employ broad-spectrum antibiotic coverage to target the most likely pathogens in patients without microbiological confirmation. However, careful consideration must be given to optimizing sample collection in order to maximize the likelihood of pathogen detection [16, 17]. At our institution, the sampling protocol aligns with current literature-based best practices. This includes:

- A pre-analytical consultation with the Antibiotic Stewardship team,
- A preoperative antibiotic-free interval of approximately two weeks when feasible,
- Collection of multiple tissue samples from all relevant intraoperative locations,
- Ensuring a minimum tissue sample size of 0.5–2 mm,
- Avoidance of superficial swab samples, and.
- Use of optimized transport media and conditions tailored to the suspected pathogens [18].

Ridder et al. have demonstrated that superficial smears often fail to correlate with deep tissue biopsy results, the latter being considered the gold standard for microbiological diagnosis. This discrepancy is especially evident in the context of common colonizing organisms such as *Pseudomonas aeruginosa*, which was identified in 45% of superficial canal swabs but only in 5% of deep biopsies [19]. A substantial body of literature across various disciplines -including orthopedics and cardiology- supports the superiority of biopsy over smear sampling for accurate pathogen detection [20, 21].

The underlying principle remains consistent in this context: when a potentially colonized surface is present, it is essential to obtain deep tissue samples to ensure accurate pathogen identification and guide appropriate antibiotic therapy. This principle is particularly relevant in regions prone to colonization, where superficial sampling may yield misleading results. Supporting this notion, a microbiology study from the field of otolaryngology also demonstrated a clear discrepancy between superficial smears and deep tissue biopsies-findings that parallel those observed in our cohort. Specially, the existing literature addresses this inconsistency in the context of tonsillar smears, where superficial cultures often failed to reflect the true pathogenic organism present in the deeper tissues [22].

The existing literature offers limited guidance, and not statistically significance association was identified in our cohort between the duration of antibiotic therapy and continuation with oral antibiotics. However, it can be reasonably postulated that oral antibiotic therapy may be effective as a continuation strategy in select patients. This assumption is primarily supported by serial MRI imaging findings. At the six-month mark, 40.7% of patients showed radiologic signs of regression. By twelve months, this figure had increased substantially to 84.6%. These findings suggest that the healing process in skull base osteomyelitis requires time and may benefit from sustained supportive therapy-including extended antibiotic treatment-as part of a long-term management strategy.

FDG PET/CT or PET/MRI can also be used as an initial and follow-up examination. According to the extant literature, there is a correlation between uptake in PET/CT and the clinical symptoms exhibited by patients [23].

What factors contribute to mortality in such a disease? In the present cohort, a significant association was observed between mortality and both advanced age and the presence of microbiological pathogen detection. Given that mortality rates reported in the literature range between 46% and 70%, these associations appear clinically meaningful [24, 25].

While age at initial diagnosis is a non-modifiable risk factor, preventative strategies and educational initiatives aimed at improving early recognition of the disease may contribute better outcomes. In contrast, the likelihood of pathogen detection-another factor associated with mortality-can be improved through rigorous adherence to optimized sampling protocols, as previously outline. Accurate microbiological identification facilitates targeted therapy, which may positively impact prognosis.

## Conclusion

Antibiotic therapy guided by antimicrobial susceptibility results obtained from tissue samples remains a cornerstone of the multimodal treatment strategy for skull base osteomyelitis. The continued refinement and expansion of this approach appear essential moving forward. This study further highlights the importance of individualized, pathogen-specific antibiotic regimes, as well as appropriate management strategies in cases where the pathogen remain undetected despite repeated diagnostic efforts.

Regular follow-up imaging, particularly at the 12-month mark, has shown encouraging signs of clinical improvement in many patients. However, these findings also reinforce the fact that the healing process is protracted, often requiring sustained therapeutic support. Long-term administration of appropriate oral antibiotics may plan an increasingly pivotal role in the management of this condition. Larger prospective, preferably multicenter studies comprising higher patient numbers are urgently needed.

Given the complexity and high mortality associated with skull base osteomyelitis, treatment should be centralized in specialized skull base centers equipped to provide all three pillars of care: presence and close collaboration with an antibiotic stewardship team for timely implementation of the adequate antibiotic therapy, experienced and well-trained surgical teams for optimal surgical interventions, and an oxygen chamber for the hyperbaric oxygen therapy.

### Limitations

A key limitation of this study is the absence of a control group receiving only surgery or hyperbaric oxygen therapy without antibiotic treatment. However, withholding antibiotic therapy in patients with skull base osteomyelitis deems unethical given the high lethality rate reported in the literature-up to 50.0% [9].

Despite this limitation, the study underscores the critical importance of accurate pathogen identification using appropriately collected specimens. This is essential for guiding targeted antibiotic therapy, which is a cornerstone of the comprehensive treatment strategy of SBO.

Because of all patients in this cohort received a combination of antibiotics, surgery, and HBO therapy, the individual contribution of each treatment modality cannot be isolated or evaluated independently. Nonetheless, the data suggest that the integrated, multimodal approach is effective in managing this complex and potentially life-threatening condition.
